# Moonlighting Proteins at the Candidal Cell Surface

**DOI:** 10.3390/microorganisms8071046

**Published:** 2020-07-14

**Authors:** Dorota Satala, Justyna Karkowska-Kuleta, Aleksandra Zelazna, Maria Rapala-Kozik, Andrzej Kozik

**Affiliations:** 1 Department of Analytical Biochemistry, Faculty of Biochemistry, Biophysics and Biotechnology, Jagiellonian University in Krakow, Gronostajowa 7, 30-387 Krakow, Poland; dorota.satala@uj.edu.pl (D.S.); aleksandra.zelazna@doctoral.uj.edu.pl (A.Z.); 2 Department of Comparative Biochemistry and Bioanalytics, Faculty of Biochemistry, Biophysics and Biotechnology, Jagiellonian University in Krakow, Gronostajowa 7, 30-387 Krakow, Poland; justyna.karkowska@uj.edu.pl (J.K.-K.); maria.rapala-kozik@uj.edu.pl (M.R.-K.)

**Keywords:** *Candida* yeast, cell wall, protein moonlighting, non-classical secretion, adhesion, plasminogen, complement system, contact system, stress protection, molecular mimicry, enolase, glyceraldehyde-3-phosphate dehydrogenase

## Abstract

The cell wall in *Candida albicans* is not only a tight protective envelope but also a point of contact with the human host that provides a dynamic response to the constantly changing environment in infection niches. Particularly important roles are attributed to proteins exposed at the fungal cell surface. These include proteins that are stably and covalently bound to the cell wall or cell membrane and those that are more loosely attached. Interestingly in this regard, numerous loosely attached proteins belong to the class of “moonlighting proteins” that are originally intracellular and that perform essentially different functions in addition to their primary housekeeping roles. These proteins also demonstrate unpredicted interactions with non-canonical partners at an a priori unexpected extracellular location, achieved via non-classical secretion routes. Acting both individually and collectively, the moonlighting proteins contribute to candidal virulence and pathogenicity through their involvement in mechanisms critical for successful host colonization and infection, such as the adhesion to host cells, interactions with plasma homeostatic proteolytic cascades, responses to stress conditions and molecular mimicry. The documented knowledge of the roles of these proteins in *C. albicans* pathogenicity has utility for assisting the design of new therapeutic, diagnostic and preventive strategies against candidiasis.

## 1. Introduction: Moonlighting Proteins—Definition and Classification

Proteins are multifunctional by nature. The largest of them are divided into distinct domains or composed of separate subunits designed to interact with a wide variety of molecular partners. Proteins can perform multiple physiologically relevant functions due to gene fusion, alternative mRNA splicing, proteolytic generation of different protein variants or promiscuous enzyme activity. However, none of these relatively well-understood molecular mechanisms underly the enigmatic behavior of a group of multitasking proteins termed “moonlighting proteins” by Jeffery in 1999 [1]. A moonlighting protein is a single polypeptide chain that has a usually evolutionally conserved function but performs an additional unrelated function via a priori unexpected interactions with non-canonical molecular targets. These extra functions often occur at an “unauthorized” subcellular or extracellular location which seems to be contrary to the classical rules of protein sorting. The existence of two or even more essentially different functions in a single moonlighting protein can also depend on the concentration of substrates or additional ligands and the oligomerization state or the formation of complexes with other proteins, often occurring without any essential structural alterations of these molecules [1,2].

The first examples of proteins displaying two fundamentally different and apparently unrelated functions were reported by Piatigorsky and coworkers [3,4], who found that individual members of a crystalline family of structural proteins of the eye lens were identical, i.e., “share the same gene”, with specific cytoplasmic enzymes. However, the term “gene sharing” proposed by those researchers was not broadly accepted because of its ambiguity. The clear-cut definition of moonlighting proteins proposed by Jeffrey [1], although sometimes criticized as too restrictive [5], precisely distinguishes this subset of much broader class of multifunctional proteins. Currently, several hundred confirmed moonlighting proteins are registered in special databases such as MoonProt Database [6] (www.moonlightingproteins.org), MoonDB [7] and MultitaskDB [8]. The rapid increase in the described cases of this type of multitasking protein in recent years suggests the possibility that many monofunctional proteins will be found in the future to moonlight.

Because of the tremendous number of combined functions (moonlighting vs. “classic”) among moonlighting proteins, additionally multiplied by the variable distribution of these functions between intracellular/extracellular compartments, a detailed classification of all moonlighting proteins will be problematic. Hence, it has been recently proposed [9] to classify them into two large groups only, thus leaving aside many individual moonlighting proteins with unique features. These two major subsets include (i) “trigger enzymes” and (ii) intracellular/secreted moonlighting proteins. The first subset represents enzymes that also regulate transcription or translation by direct binding to DNA or RNA, or by binding to other proteinaceous translation or transcription factors [10]. The second group, the largest within the entire class of known moonlighting proteins, includes intracellular proteins: ‘housekeeping’ enzymes, chaperones, translation factors, DNA-binding proteins and many others that are secreted and either reside attached to the cell surface, acting as receptors for soluble proteins or small molecules, or function in the fluid phase, often for intracellular signaling [9].

The intracellular/secreted moonlighting proteins have been found in organisms from all kingdoms and this type of protein moonlighting has been most widely observed and best characterized in pathogenic bacteria (for reviews see: [11,12,13,14]). Bacteria commonly non-conventionally secrete cytosolic proteins and expose them at their cell surface to interact with the host. Many of these proteins have been confirmed to play important roles in biofilm formation, adhesion to the host cells and extracellular matrix, host tissue invasion, infection and virulence. Some of these factors, either cell-bound or soluble, can interfere with host defense mechanisms via interactions with, or by affecting, components of the host immune system. 

Although obviously similar pathogenic mechanisms involving intracellular/secreted moonlighting proteins must be used by eukaryotic pathogens, the phenomenon of moonlighting proteins has been recognized to a much lesser extent in these organisms than in bacteria. This issue has been covered by a small number of recent reviews [15,16]. A comprehensive overview of protein moonlighting in yeast has also been published [17] but predominantly presents data on the non-pathogenic baker’s yeast *Saccharomyces cerevisiae*. In our current review, therefore, we critically summarize the current state of knowledge on cell surface-exposed moonlighting proteins in the opportunistic pathogen *Candida albicans*, one of the most common fungal pathogens in humans and in several other closely related and medically important *Candida* species. 

## 2. Atypical Proteinaceous Components of Candidal Cell Wall

The cell wall of *Candida* yeast is an external envelope of the fungal cell that determines its shape and strength. It is both a rigid protective shield and a dynamic interface to the external milieu within the human host. Undergoing a constant remodeling upon interactions with various host molecular targets, the candidal cell wall senses any changes in its environment and immediately sends signals to the cell to respond to these changes during host colonization and infection [18].

**Figure 1:**
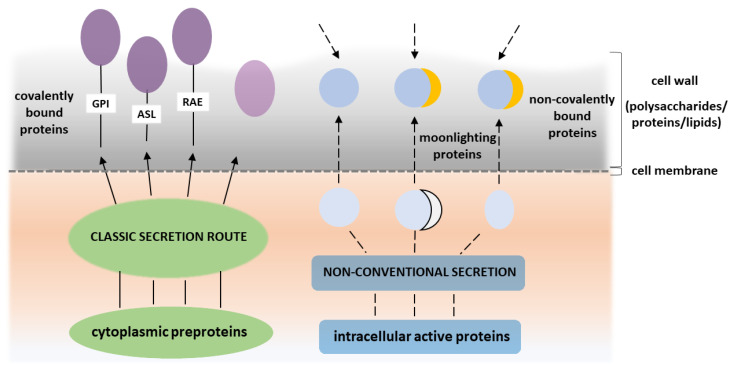
Major classes of proteins present at the *C. albicans* cell wall. Cytoplasmic preproteins that possess signal peptide pass the classical secretory route and emerge at the cell surface as “typical” cell wall proteins that are covalently anchored via three major linkages―GPI, ASL and RAE―but can also exist as loosely associated with the cell wall polysaccharide skeleton. In contrast, “atypical” proteins, despite the lack of a signal for the classical secretion pathway, appear at the cell wall due to an unconventional secretion or re-adsorption. Those that take on new functions at the cell surface are called moonlighting proteins.

The cell wall of *C. albicans* is a multilayered complex structure (for recent reviews see: [19,20,21,22]). The inner layer is composed of chitin and a network of β-1,6-glucans and β-1,3-glucans. The outer layer contains mainly proteins of various types [22,23,24]. Although constituting less than 10% of the *C. albicans* cell wall, proteins perform many important functions in this microbe, including participation in the initial colonization of epithelial cells and the further development of host infection [23,25,26,27,28,29]. The proteome of the yeast cell wall is variable and depends primarily on the morphological form and culture conditions [23,30,31,32]. Since the seminal review of Chaffin in 2008 [22], yeast cell wall proteins have been classified into two types (Figure 1), i.e., ‘typical’ and ‘atypical’ [22,23,33].

Precursors of typical cell wall proteins have a signal sequence at the N-terminus and are transported to the cell surface via a classical secretion route. Those proteins are usually covalently linked to the cell wall via various different bonds. The first of these bond types, and the most widely found, is a glycosylphosphatidylinositol (GPI) anchor which attaches, e.g., the well-characterized agglutinin like sequence family (Als) of proteins, putative GPI-anchored family (Pga) of proteins, hyphal wall protein (Hwp1), enhanced adherence to polystyrene 1 (Eap1) protein and chitinase 2 (Cht2). These proteins play essential roles not only in the phenomena of adhesion, invasion of epithelial cells, biofilm formation and aggregation of *C. albicans* cells, but also on the remodeling of candidal cell wall (for recent reviews see: [22,23,34,35]). The second type are alkali-sensitive linkages (ASL) that are characteristic of Pir proteins (proteins with internal repeats) [26,27,36]. The third type of bond is a reducing agent-extractable (RAE) interaction based on disulfide bridges, exemplified by the proteins of the hyphally-regulated family (Iff2/Hyr3) [23,37]. There is also a group of proteins that are secreted to the surface via the classical pathway as the signal peptide, for this process is harbored by their precursors. They can then get trapped inside the cell wall or at the cell surface, remaining loosely associated with the cell wall polysaccharide skeleton. Examples of such proteins include 1,3-β-glucosyltransferase (Bgl2) and exo-1,3- β-glucanase (Exg1) [23,38,39]. Some of the soluble secreted aspartic proteases (Saps) may also present this behavior [40]. 

Typical cell wall proteins cover *C. albicans* cells and perform many important functions on the cell surface. Some serve as adhesins (Als-family, Eap1 andEcm33) that contribute to yeast adhesion to the host cells or abiotic surfaces. Others are proteins associated with cell wall biogenesis and remodeling (Cht1-2, Crh11-12, Exg2, Kre1, Pga4, Pht1-2, Pir1, Spr1, Ssr1, Utr2, Csa1, Rbt5 and Pga10) that maintain the proper cell wall composition. Yet, another group includes enzymes (Cht2, Plb5, Sap9-10 and Sod4-6) that play a crucial role in the invasion of host cells and in cell wall remodeling during this pathogenic event [22,39]. 

Atypical proteins that do not have a signal for the classical secretion pathway and remain non-covalently associated with the cell wall include two groups. One is represented by proteins that perform the same function both inside and outside the cell so that they do not fulfill the restrictive Jeffery’s definition of moonlighting proteins [1]. An example could be heat-shock protein 90 (Hsp90), that retains the ability to perform unique functions even at unusual cellular locations [41]. These proteins may possibly have an additional function, but this has yet to be determined. Hence, the intracellular Ssa chaperones/stress protectants that have been frequently detected at the candidal cell wall [42,43,44] still chaperone at that site, but a moonlighting adhesin function has recently been suggested for these proteins, at least in some *Candida* species [45,46]. In contrast, the second group is mainly represented by proteins that perform enzymatic functions in the central metabolic pathways, but that play new and unusual roles when located at the cell wall. Once this role is experimentally confirmed, a multifunctional protein can be considered to have moonlighting actions. Generally however, their presence on the cell wall of *C. albicans* is considered unusual [23,47,48,49,50]. 

Given that the numerous moonlighting proteins detected at the surface of *C. albicans* are intracellular factors, the question arises about the mechanism of their transport to the external milieu. Since these proteins are not equipped with an N-terminal signal for extracellular secretion, they cannot readily participate in the classical secretory pathways through the Golgi apparatus. Although it is possible that these predominantly cytoplasmic proteins are sourced from damaged fungal cells located in the same environment as their viable growing counterparts and are simply re-adsorbed to the cell surface [51,52], they are more likely to have been subjected to a non-classical secretion mechanism and actively transferred to the cell wall. This may be corroborated by the fact that the changes in the composition of the cell surface-connected moonlighting proteins are often an effect of the cell response to specific environmental factors and they thus improve the adaptation of microbial cells to new and demanding conditions [53,54,55,56,57]. 

One of the most frequently postulated mechanisms for the secretion of *C. albicans* moonlighting proteins is their transport outside the cell as a cargo of extracellular vesicles (EVs) [58,59,60,61]. Moreover, other mechanisms of non-classical secretion have been proposed for the extracellular transport of surface-exposed moonlighting proteins for numerous eukaryotic organisms, although they still require detection and analysis in *Candida* yeasts. Such putative secretory routes include the direct translocation of proteins through the cell membrane by dedicated membrane-associated transporters, modulation of the activity of plasma membrane flippases, membrane flipping together with the adhered cytoplasmic proteins exhibiting the affinity for phospholipids and the escape of cytoplasmic proteins during cell division through the fragmentation zone engaging a lock-type mechanism [62,63,64,65,66]. In the case of some human and bacterial moonlighting proteins, it has been suggested that particular post-translational modifications, such as 2-phosphoglycerate-dependent automodification, rhamnosylation or monomethylation, might support their extracellular transport or membrane localization [67,68,69]. This issue has yet to be investigated for *C. albicans* proteins however.

Recent proteomic studies have repeatedly revealed the presence of numerous putative moonlighting proteins in the candidal cell wall proteome [24,39,70,71,72], or in its subsets selected by special treatments such as pre-adsorption to host proteinaceous factors [73,74,75] or cell surface shaving by trypsin [24,56,72,76,77]. Classified according to their original intracellular function, the moonlighting proteins detected in the cell wall of *C. albicans* include:

- enzymes involved in evolutionally conserved central metabolic pathways such as:
  ○ glycolysis and/or gluconeogenesis (phosphofructokinase (Pfk1), fructose-bisphosphate aldolase (Fba1), phosphoglycerate mutase (Gpm1), glyceraldehyde-3-phosphate dehydrogenase (Tdh3), enolase (Eno1) and pyruvate kinase (Cdc19));
  ○ fermentation (alcohol dehydrogenase (Adh1));
  ○ the pentose phosphate pathway (6-phosphogluconate dehydrogenase (Gnd1));
  ○ the Krebs cycle (aconitase (Aco1) and citrate synthase (Cit1));
- factors associated with protein synthesis such as:
  ○ ribosomal proteins (including Rpl3, Rpl5, Rpl9B, Rpl12, Rpl13, Rpl18, Rps22A and Rps31);
  ○ elongation factors (elongation factor 2 (Eft2), elongation factor 3 (Eft3), translation elongation factor 1-alpha (Tef1) and elongation factor 1-beta (Efb1));
- chaperones (Ssa1, Ssa2, Ssa4, Ssb1, Ssc1, Ssd1, Ssz1 and Kar2);
- enzymes involved in redox homeostasis (superoxide dismutase (Sod3), glutathione reductase (Glr1), peroxiredoxin (Tsa1)); [24,30,33,39,70,72,73].

Moonlighting proteins have also been detected at the cell surface of other *Candida* species such as *C. tropicalis, C. parapsilosis* and *C. glabrata* [56,78]. Most of the proteins detected in those studies were homologous to the moonlighting proteins found in *C. albicans* such as Eno1, Gpm1, Tdh3, Fba1, Adh1 and triosephosphate isomerase (Tpi1). In addition, the elongation factors (Tef1, Efb1, Efb3) and chaperons (Ssa2, Ssb1) have been detected at the cell wall of non-albicans *Candida* species [56,73,74,78,79,80,81,82]. 

As described in the following sections, many of these atypical candidal cell wall proteins have new experimentally supported moonlighting functions. For others, however, the alternative functions acquired on the fungal cell surface await to be validated to substantiate their classification as moonlighting proteins. 

## 3. Acquired Functions (Moonlighting) of Atypical Cell Wall Proteins of *Candida* Spp.

### 3.1. Adhesion

The essential attribute of moonlighting proteins is an ability to bind to host proteins and cells. These interactions have been most widely studied in terms of their contribution to the adhesion to host tissues and the interference with host regulatory proteolytic cascades. Representative examples of these two major of moonlighting functions in *C. albicans* are briefly presented in Table 1.

At the initial stages of infection, the task of pathogen surface proteins is to maintain physical interactions with human epithelial cells. Damage to the outer mechanical protective layer results in the presentation of the basal membrane and extracellular matrix (ECM) components, such as fibronectin (FN), laminin (LAM) and vitronectin (VTR), that, when exposed, provide additional sites for pathogen attachment. The ability to recognize and bind to ECM components was clearly shown to correlate with the pathogenicity of *C. albicans* strains [89].

The first cell surface moonlighting protein of *C. albicans* to be identified was Tdh3, an enzyme involved in the glycolysis pathway that had a secondary role at the surface of yeast as an ECM-protein binding ligand. The use of polyclonal antibodies confirmed its role in the interaction with FN and LAM [83]. It was shown, using a ligand western blotting assay, that purified cytoplasmic Tdh3 also acts as a ligand for FN and LAM. This reflects the frequent situation that the newly acquired function of a moonlighting protein is not necessarily associated with a structural modification of its cytoplasmic precursor [83]. A similar function in recognizing ECM components was shown for Adh1. Based on the screening of a cDNA library of *C. albicans* yeast cells with polyclonal antibodies against human integrins, Adh1 was qualified as a protein that plays a role in interacting with VTR and FN [89]. 

It is generally postulated that moonlighting proteins indirectly support pathogen binding to host cells, thereby facilitating the colonization process and promoting the subsequent invasion of the host organism. An example of such a protein is Eno1, which performs its primary function in the glycolysis pathway but has been repeatedly detected at the surface of numerous organisms, including non-pathogenic microbes [11,12,13]. In *C. albicans*, Eno1 was shown to be an essential adhesin for mouse intestinal endothelial cells. A preincubation of the intestinal mucosa with anti-Eno1 antibodies or recombinant Eno1 resulted in a decrease in the adhesion of *C. albicans* by 48%–70% [28]. Moreover, it was demonstrated that extracellular Eno1 is presented on the biofilm-forming cells [28,91] and plays a role in adherence to silicone and polyvinyl chloride—biomaterials that are used to produce medical elements, such as venous catheters, valves or orthopedic prostheses. Thus, it was suggested that Eno1 participates in biofilm adhesion and formation [28,92]. Other cytoplasmic enzymes, glycerol-3-phosphate dehydrogenase (Gpd2) and Gpm1, both involved in carbohydrate metabolism, were qualified as factors that bound directly to human keratinocytes (HaCaT) and human umbilical vein endothelial cells (HUVECs) [29,90]. The authors of those reports suggested that Gpd2, detected at the surface of yeast cells and hyphae, may also bind to extracellular matrix components that are released by human endothelial cells as bound Gpd2 was not only detected on human cell monolayers, but also within the extracellular space [90]. Using an enzyme-linked immunosorbent assay, Gpm1 was identified as a VTR- and FN-binding protein but was found to be unable to interact with LAM and collagens. Notably, however, the interaction with FN was weaker, indicating a predominant role of VTR in Gpm1 binding. Interestingly, no ability to interact with pre-monocytic cell line U937 was demonstrated for either Gpd2 or Gpm1, suggesting some ligand preferences for these moonlighting proteins [29,90]. 

Both the adhesion to and subsequent invasion of host cells are critical to the survival of the fungus in the infected host. The active invasion of host epithelial cells by *C. albicans* is based on the endocytosis induced by the interaction of the human cadherins with proteins exposed at the surface of the pathogen [93,94,95]. The Ssa1 chaperone that belongs to the heat shock protein 70 (Hsp 70) family, was reported to be exposed at the yeast cell wall and to be sufficient to induce endocytosis. Latex beads coated with recombinant Ssa1 were found to be rapidly endocytosed by both oral epithelial and endothelial cells, thus suggesting the crucial role of this protein in virulence [45]. Moreover, pull-down tests using recombinant proteins and a yeast-two hybrid assay found that both Ssa1 and Ssa2, another member of the Hsp70 family, acted as receptors for the salivary histidine-rich basic antifungal peptide, histatin 5 (Hst5), with Ssa2 exhibiting a significantly stronger interaction than Ssa1 [42].

For medically relevant non-albicans *Candida* species, several studies have reported a role of cell surface moonlighting proteins in the adhesion phenomena. Based on the results of affinity chromatography experiments, proteinaceous partners present at the *C. tropicalis* and *C. parapsilosis* cell wall and capable of interacting with ECM proteins were suggested. Five proteins―malate synthase (Mls1), Eno1, Fba1, transaldolase (Tal1) and Eft2―and eight further proteins―Eno1, Gpm1, Mls1, amidase (Amd2), glucose-6-phosphate isomerase (Pgi1), Gnd1, Eft2 and transketolase (Tlk1)―that had a putative binding affinity to FN, LAM and/or VTR were identified at the cell surface of *C. tropicalis* and *C. parapsilosis*, respectively [74]. 

### 3.2. Interactions with Host Homeostatic Proteolytic Cascades

The ability of pathogen surface proteins to interact with the components of the host’s homeostatic/hemostatic proteolytic cascades, i.e., the fibrinolysis, complement and kallikrein-kinin systems, contributes to the invasion of host tissues and facilitates the acquisition of nutrients. Thus, the interaction of surface moonlighting proteins with serum proteins is a newly acquired function that contributes to the virulence of *Candida* strains but should be considered separately from their involvement in the adhesion phenomena. Moreover, a potential dual significance of the pathogen interactions with host homeostatic systems should be kept in mind because, depending on the infection context, pathogens can benefit both from system hijacking via the pathogen surface-catalyzed activation and system inhibition via the sequestration of critical host proteins through their tight attachment to the pathogen surface. 

One of the key infection-relevant mechanisms described for many pathogenic bacteria and fungi is an ability to interact with soluble human plasminogen (HPG) [96,97]. HPG is a precursor (zymogen) of plasmin―a serine protease that plays an essential role in fibrinolysis by breaking down fibrin clots. HPG can be activated by factors such as tissue plasminogen activator (tPA) or urokinase plasminogen activator (uPA). Notably, however, it was suggested that the binding of HPG via receptors exposed at the surface of the pathogen can also result in its transformation into plasmin in a mechanism not controlled by the host. The resulting active protease, still associated with the pathogen surface, can degrade components of the basement membrane or extracellular matrix, but also activate other important proteinases, e.g., those involved in tissue remodeling [98,99]. The triggering of those proteolytic events by HPG activation can open up new migration routes for the pathogen through the host tissue. Hence, it can be hypothesized that pathogen surface proteins capable of HPG binding can be considered to be important virulence factors.

HPG has been shown to bind to multiple proteinaceous components of the *C. albicans* cell wall. Several proteomic studies have indicated that 10 moonlighting proteins―Gpm1, Adh1, Eno1, Gpd2, Tdh3, peroxisomal catalase (Cat1), Tsa1, phosphoglycerate kinase (Pgk1), Tef1 and Fba1―possess a high affinity for HPG [77,84,88,90]. It was also shown in another report that the candidal invasion of microvascular endothelial cells of the human brain was induced after the binding of Eno1 to HPG, which facilitated the crossing of the blood-brain barrier in vitro [85]. Moreover, HPG, when bound to Eno1, Gmp1 or Gpd2 can still be activated by uPA, and the resulting plasmin exerts proteolytic action on fibrinogen and extracellular matrix proteins [86,88,90]. It was suggested also that HPG binding to surface-exposed moonlighting proteins results in a concentration of proteolytic activity in very close proximity to the yeast cell wall, an effect that may explain how the fibrinolytic activity supports tissue penetration by *C. albicans* cells [86].

Moonlighting proteins can also interact with the complement system, the activation of which is aimed at recognition of the pathogen by immune effector cells and its subsequent opsonization, finally resulting in direct pathogen killing via an attack on the cell membrane or activation of the phagocytosis pathway. Bacteria and fungi have developed mechanisms, however, that prevent the activation of the complement system, e.g., by recruiting its protein components at the pathogen surface [77,100]. In the case of *C. albicans*, it has been shown that Gpm1 and Gpd2 can interact with components of alternative complement pathway: factor H (FH) and factor H-like binding protein 1 (FHL-1) [88,90]. Interestingly, Gpm1-attached plasma factors retain their biological activity and show cofactor activity toward the cleavage of C3b [88]. It was also shown that a high-affinity glucose transporter 1 (Hgt1) blocks the activation of the complement system. Another study, using a homozygous *hgt1Δ/Δ* deletion mutant, confirmed the recruitment of FH at the surface of *C. albicans* via Hgt1 and demonstrated its ability to interact with the C4b binding protein, which is the main regulator of the classical and lectin pathway [101]. It was also suggested in a further report that factor H attached to the surface of endothelial cells (HUVECs) and keratinocytes (HaCaT cells) could act as a bridge for the interactions between Gpd2 and host cells [90]. Due to this effect, the pathogen attached to the epithelial cells remained latent for the immune system and could begin the process of active invasion, e.g., with the previously mentioned Ssa1 chaperone.

Interactions of *C. albicans* moonlighting proteins have also been described for the so-called contact system, i.e., the plasma contact-activated kinin-generating system, also known as the kallikrein/kinin system, that contributes to the host inflammatory response during infection. The main components of this proteolytic cascade are zymogens of the serine proteases, i.e., coagulation factor XII (FXII) and plasma prekallikrein (PPK) and a non-enzymatic factor high molecular weight kininogen (HK), which is a proteinaceous precursor of universal proinflammatory mediators called kinins. The contact system acts as a double-edged sword during infections. On the one hand, vasoactive and pro-inflammatory kinins cause vasodilatation, thereby increasing the permeability of blood vessels and the chemotaxis of immune cells to the site of infection. On the other hand, the increased blood inflow facilitates the acquisition of nutrients necessary for pathogen growth [100,102,103].

Affinity chromatography experiments have identified the main *C. albicans* moonlighting proteins capable of interacting with the components of contact system. Among these, Eno1, Gpm1, Eft2 and Tpi1 were found to interact with HK, FXII and PPK. Another seven proteins―Gnd1, hexokinase 2 (Glk4), Tsa1, Sod3, 2-oxoglutarate dehydrogenase complex E2 component, Pgi1 and glutathione reductase (Glr1)―showed binding to one or two plasma proteins [73,87]. In addition, four moonlighting proteins―Eno1, Gmp1, Tpi1 and Pgi1―were purified and their interaction with human contact factors was characterized in terms of kinetic and thermodynamic parameters [73]. Interestingly, Tpi1 presented the highest affinity to HK and FXII, whilst Pgi1 showed the highest specificity for ligands because it could only bind to PPK [73].

Some moonlighting proteins of non-albicans *Candida* species have also been reported to interact with host homeostatic proteolytic cascades. In *C. parapsilosis*, Eft2, Mls1, Pgi1, Gnd1, Tal1, acetyl-CoA synthetase (Acs1), phosphoenolpyruvate carboxykinase (Pck1) and NAD-aldehyde dehydrogenase (Ald5) showed an affinity for HPG and Ssa2, and Gnd1 was shown to bind both HPG and HK. In *C. tropicalis*, seven surface moonlighting proteins―Gpm1, Eno1, Eft2, Gnd1, Pgk1, Tal1 and fructose-1,6-bisphosphatase (Fbp1)―were found to be capable of interacting with HK [46,79].

### 3.3. Stress Protection

The external stress factors that a pathogen must cope with within various niches in the host organism include temperature changes, osmotic changes, dehydration, oxidative stress, host immune responses, variable oxygen availability and nutrient deficiency. All these stressors drive the expression of virulence factors that enable survival under adverse conditions. Among these factors, the cell-surface exposed moonlighting proteins can contribute to the pathogen’s defense against the cellular stress. 

In several proteomic studies it was shown that *Candida* spp. grown in a liquid medium and in the form of biofilms altered the expression and amount of moonlighting proteins expressed on its surface―Eno1, Tdh3, Fba1, Pgk1, Gpm1, Tpi1 and Ssa2―in the presence of hydrogen peroxide and menadione [54,55,104]. Moreover, Adh1, Eft2, Ssa1, Ssb1, F_1_F_0_-ATPase complex (Atp2) and cadmium induced protein (Cip1) were found to be upregulated under conditions of high hydrogen peroxide, salt and cadmium levels [105]. Studies using a *∆tsa1* mutant further showed that the exposure of Tsa1 on the surface of *C. albicans* hyphae protected the fungal cells from damage under conditions of oxidative stress [106]. Due to their likely location in the outermost layers of the cell wall, moonlighting proteins may therefore be the first line of defense against reactive oxygen species generated in vivo by the phagocytic cells of the immune system, although the underlying mechanisms are still unknown. One possible mechanism, yet to be experimentally verified, could be that yeast-surface localized moonlighting proteins “discharge” reactive oxygen species, accumulating oxidative modifications such as S-thiolation, carbonylation or tyrosine nitration that are detectable under the presence of hydrogen peroxide for highly homologous proteins such as Eno1, Tpi1, Tdh3 and Fba1 of the non-pathogenic yeast *S. cerevisiae* [107,108,109,110]. It was suggested also that *C. albicans* Eno1 plays a role in osmotic protection owing to its transglutaminase activity, catalyzing protein modifications, such as cross-linking, amine incorporation and deamination [111].

The response of *C. albicans* to thermal stress seems also to engage cell-surface exposed moonlighting proteins. Such a function was suggested for 5-methyltetrahydropteroyltriglutamate-homocysteine methyltransferase (Met6) [112] and for Ssa1 and Ssa2 proteins, members of Hsp70 family, which were shown to contribute to the adaptation of *C. albicans* to temperature variations [104]. The role of Ssa1 was suggested to rely on its interaction with a transmembrane adhesin-like protein (Msb2) from the mucin family, an event that triggers a cellular response via the CEK mitogen-activated proteins kinase (MAPK) pathway [113]. Thus, Ssa1 can act as a sensor that regulates the cellular response to protect the cell from harmful environmental conditions. Some additional related evidence comes from studies on *S. cerevisiae* in which the expression of several heat shock proteins changed after exposure to high-temperature stress. One of them was Hsp48, which is actually the protein product of the *ENO1* gene regulated by the heat shock resistance gene (*HSR1*) and associated with temperature tolerance [114]. Hence, it is not so unexpected that *C. albicans* Eno1, showing 88% similarity to the amino acid sequence of the *S. cerevisiae* enzyme, showed a high thermostability and was suggested to be associated with thermal stress [115,116].

Observations that *C. albicans* upregulates many genes encoding enzymes involved in glycolysis and fermentation under hypoxia may indicate that moonlighting proteins can help the yeast to adapt to variable oxygen availability [117,118]. It was recently confirmed that *C. albicans* produces Eno1 in increased amounts upon contact with the periodontal bacterial pathogen *Porphyromonas gingivalis* under anaerobic conditions [119]. The change in the expression of *C. albicans* Eno1 under hypoxic conditions may indicate that this protein protects yeast cells during the cellular response to hypoxic stress by promoting anaerobic metabolism.

### 3.4. Molecular Mimicry

Molecular mimicry is another mechanism considered to be essential for the pathogenicity of microorganisms. The basis of this phenomenon is the similarity of some cell surface-exposed pathogen antigens to host proteins which assist the pathogen to evade the host immune response. In other words, some microbe surface factors mimic human antigens. Thus, the proteins located in the uppermost layer of the cell wall form a shield against the host defense mechanisms that target invasive pathogens and thereby allow these unrecognized pathogens to migrate deeply into the host tissue and develop the infection [120]. 

A contribution of moonlighting proteins to the hypothetical protective coat that renders the pathogen surface more compatible with the host was first suggested for some bacteria. For example, the *Klebsiella pneumoniae* enolase was shown to present short C-terminal domain sequences that are identical to human muscle enolase [121] and the ribosomal proteins P1, P2 and B13 of the *Trypanosoma cruzi* parasite present an epitope that mimics cardiac myosin [122,123]. 

The first described *C. albicans* mimetic protein was an integrin analogue. Based on the analysis of clinical strains, this surface-exposed integrin analogue was shown to inhibit fungal opsonization and phagocytosis due to competition with the complement type 3 receptor (CR3) (also known as CD11b/CD18) that presented on peripheral blood neutrophils [124,125]. Upregulated expression of the integrin analogue also contributed to a significant increase in yeast cell adhesion to human epithelial and endothelial cells. Since the described integrin analogue responded to glucose, it could have been mimicking the Hgt1 described later by another group [101]. As mentioned above, Hgt1 acts as an inhibitor of the complement system. Analyses carried out using a homozygous *hgt1∆/∆* deletion mutant revealed that *C. albicans* cells lacking this protein on their surface were unable to form rosettes with complement coated erythrocytes, suggesting that these cells also had a significantly reduced expression of the CR3 receptor analogue [101]. 

According to the molecular mimicry hypothesis, the immune system should not elicit a response against pathogen proteins that present epitopes identical to host proteins. Conservative moonlighting proteins displayed at the surface of *C. albicans* seem to be ideal for this mimicking task [120,126]. Notably also, molecular mimicry by pathogenic fungi can also provoke the development of autoimmune diseases.

Autoimmune polyglandular syndrome type I (APS I) was shown to be associated with chronic mucocutaneous candidiasis and the fungal antigen underlying this disease was found to be enolase in 80% of the affected patients. Because of the high similarity between fungal and human enolases, an antibody cross-reaction can occur, thus explaining the relationship between candidiasis and the development of APS I as a consequence of mimicking the epitopes of these two proteins [127,128]. Interestingly, antibodies against citrullinated α-enolase have been identified in the autoimmune response of 40%–60% of patients with rheumatoid arthritis [129,130,131]. It was thereafter assumed that citrullination―a post-translational modification of proteins by peptidylarginine deiminases (PAD)―might be one of the mechanisms generating epitopes previously hidden from the immune system [130,131,132]. It was also demonstrated that citrullinated α-enolase antibodies cross-react with bacterial enolases [130]. In addition to host endogenous PADs, enzymes synthesized and released by an anaerobic periodontal bacterium *P. gingivalis* (PPAD) may also appear in the host organism. For *C. albicans*, it was shown that seven moonlighting proteins Eno1, Adh1, elongation factor 1-alpha 1, Tdh3, heat shock proteins Ssa1 and Ssa2 and ATPase 1 of the plasma membrane could be citrullinated by PPAD [133,134]. The ability to citrullinate fungal moonlighting proteins may affect the disclosure of epitopes that have no immune tolerance, which consequently induces antigen production and autoimmunity.

### 3.5. Atypical Enzymatic Activity

Changes to the concentration of substrates and ligands, the state of oligomerization, expression of other proteins and post-translational modifications can affect the role of moonlighting protein under given conditions [1]. In most studies to date, the moonlighting functions of *C. albicans* intracellular proteins at the cell surface have been reported to be non-enzymatic. However, in many organisms, protein multi-functionality can also include multiple catalytic activities. As examples of this, human Tdh3 not only functions in the glycolysis pathways, but also has phosphotransferase activity, human Pgk1 participates in the process of angiogenesis due to the activity of disulfide reductase, and bacterial Pgk1 exhibits Tpi1 activity due to the identical N-terminal sequence [135,136,137]. Unfortunately, this issue has not yet been extensively investigated in *Candida* yeasts. It was recently reported that one of the essential proteins responsible for transglutaminase (TGase) activity on the *C. albicans* cell wall is the major multifunctional protein, Eno1 [111]. TGases are multifunctional enzymes, usually dependent on calcium ions, which are involved in post-translational modifications such as protein cross-linking, amine incorporation, and deamination. These enzymes play an important role in many biological processes, such as cell growth and differentiation, maintaining tissue integrity and stabilizing the extracellular matrix [138]. For *C. albicans* and *S. cerevisiae* yeasts it was shown that active TGases are primarily present in cell wall fractions. Due to the ability to form covalent cross-links between proteins and chitin and/or glucan, yeast TGases mediate the organization of the cell wall structure, enabling protein incorporation [139,140]. Inhibition of TGase activity by cystamine significantly affects the *C. albicans* cell phenotype, such that they became unable to form hyphae [111,139]. Experiments using recombinant *C. albicnas* Eno1 have confirmed that in addition to its classical activity associated with the glycolysis pathway, Eno1 also has TGase activity. Because antibodies against Eno1 were only able to block the enzyme’s basal activity, it was suggested that the dual catalytic function of Eno1 is associated with the presence of two independent catalytic sites within the molecule [111]. 

Double enzymatic activity has also been reported for *C. albicans* Adh1, which catalyzes the conversion of acetaldehyde to ethanol and also participates in the NADH-dependent catalysis of methylglyoxal (MG) oxidation and reduction [141]. MG is a reactive dicarbonyl compound formed mainly as a byproduct of metabolism on the glycolytic pathway. The accumulation of MG in the cells of microorganisms has been shown to cause damage to cell systems, which results in cell growth inhibition. To prevent this, MG is detoxified with methylglyoxal reductase [141,142]. Interestingly, methylglyoxal reductase activity (NADH-dependent) was also reported for *S. cerevisiae* Adh1 [143]. Whether this activity can be performed by Adh1 at the candidal cell surface, and thus be classified as a moonlighting function of this enzyme, remains to be experimentally tested. 

## 4. Confirmed Roles in Candidal Virulence and Pathogenicity

Considering the frequent involvement of fungal surface-localized moonlighting proteins in the interactions between *Candida* species and the human host, and their relevant contribution to the adaptation of fungal cells to the constantly changing and adverse environmental conditions in the host organism, their general role in the virulence of these pathogens cannot be underestimated. For several moonlighting proteins, it has been shown that a lack of their production by the fungal cells, corresponding also with the loss of their surface exposure, might lead to a significant decrease in *C. albicans* virulence, as has been repeatedly demonstrated in animal model studies. However, it must also be noted that they are often proteins of key importance for cellular biochemistry and carbon assimilation. Hence, the observed effects can often just be illustrative of the overall phenomenon associated with a deficiency of any enzyme of a particular metabolic pathway [144,145]. Nevertheless, for several particular moonlighting proteins, for which an important role in host interaction via their expression on the fungal cell wall has been confirmed, these effects might be closely interrelated.

One of the moonlighting proteins contributing significantly to the virulence of *C. albicans* has been proven to be Eno1. An *eno1/eno1* null mutant strain, which admittedly could not grow on glucose-containing media but could be propagated using non-fermentable carbon sources, demonstrated attenuated hyphal formation, increased drug susceptibility and diminished infectiveness [146]. The overall effect of the lack of Eno1 both inside the cells, and consequently also at their surface, caused significant changes in the biology and virulence of this *C. albicans* null mutant. The important role of Eno1 in inhibiting the formation of germ tubes and hyphal forms, a crucial requirement for achieving full virulence by *C. albicans* [147,148], was also demonstrated [146]. Moreover, the deletion of the *ENO1* gene induced a notable increase in the susceptibility of fungal cells to amphotericin B and miconazole and also, to a slightly lesser extent, to fluconazole and voriconazole. Most importantly, however, the lack of enolase resulted in a significantly decreased infectivity of *eno1/eno1* null mutant cells, compared to the wild type *C. albicans* strain SC5314 or *eno1/eno1::ENO1* rescued cells in a mouse intravenous infection model [146].

Fba1, another key cytoplasmic enzyme in the glycolysis pathway, has also been found to be an important virulence factor for *C. albicans* [149]. The depletion of the *FBA1* gene inhibits the growth of the *fba1/MET3-FBA1* mutant. These mutant cells remain viable but show a partially reduced pathogenicity in a mouse model of systemic candidiasis, as evidenced by the lower fungal burden in the kidneys compared with the heterozygous *MET3-FBA1/FBA1* or wild strains [149]. 

Another cytoplasmic enzyme, Adh1, that is also present at the *C. albicans* surface is not only crucial for the physiology of these fungal cells through its involvement in fermentation, but also significantly affects their virulence. Deletion of the *ADH1* gene significantly lowers the host infection ability of these cells, as confirmed in three independent infection models: *Galleria mellonella* and *Caenorhabditis elegans* invertebrate models and a mouse model of disseminated candidiasis [150]. In all of these models, infection by the *adh1Δ/Δ* knock-out mutant resulted in a considerably prolonged survival time compared with the reconstituted knock-out strain (*adh1Δ/ADH1*) or the wild type *C. albicans* strain SC5314. In addition, a noticeably lower fungal burden was evident in the liver and kidneys in the latter model infected with the strain harboring the *ADH1* gene deletion [150]. This reduction in the pathogenicity of *C. albicans* may be related to defects in filamentation caused by the lack of Adh1 in the fungal cells because it is also associated with the inhibition of mitochondrial oxidative phosphorylation that disrupts the cAMP-PKA pathway regulating the morphological transition [150,151,152]. In prior studies [153], however, it was demonstrated that Adh1 activity may inhibit the formation of a fungal biofilm due to the biocidal effects of the ethanol produced by this enzyme from acetaldehyde. Hence, the overall regulatory effect of Adh1 during the formation of filamentous forms and biofilm development, and thus virulence, is quite complex and requires further elucidation. As these effects were also demonstrated for soluble, extracellular Adh1, the evidence indicates that this moonlighting protein plays a role in the stimulation of human monocytes from the THP-1 cell line to differentiate into macrophage-like cells, enhancing their ability to phagocytose and kill *C. albicans* cells and to increase cytokine production [154].

It has also been demonstrated that the lack of production in the cell of a protein from another group not related to carbohydrate metabolism, i.e., the heat shock protein Ssa1, also dramatically reduces the virulence of *C. albicans*. The cells of the knock-out mutant strain *ssa1Δ/Δ* are significantly less invasive than those of the wild type strain SC5314 or the *ssa1Δ/Δ::SSA1* complemented strain in mouse models of oropharyngeal and hematogenously disseminated candidiasis. In addition, the fungal burden in the kidneys, brain and liver is lower with the strain deprived of Ssa1. However, this effect was found not to be associated with a defect in the formation of hyphal forms [45]. Furthermore, as described above for the surface-connected glucose transporter Hgt1, the ability of Ssa1 to bind factor H (FH), the inhibitor of the complement alternative pathway, and thus to inhibit molecular mimicry also has a direct substantial impact on the pathogenicity of fungi. The absence of this protein at the cell surface of *C. albicans hgt1Δ/Δ* null mutant caused the cells of this strain to lack a coating with FH and be phagocytosed and killed more often by isolated human polymorphonuclear neutrophils than the cells of the wild type strain SN152 [155]. All noticeable effects of the reduction in fungal virulence after deprivation of the cells of each of these individual proteins described above corroborates their direct and significant impacts on the invasiveness of *C. albicans*.

Locating and gathering several enzymes involved in basic cell metabolism, including the glycolysis pathway or gluconeogenesis, at the cell surface may also contribute to an increase in the pathogenic potential of microorganisms, especially those forming organized communities, i.e., biofilms, at the site of infection. Several previous reports have indicated that numerous moonlighting proteins are present at the cell surface of fungal cells forming mono- or mixed-species biofilm, or are embedded in the biofilm matrix, a location that can be achieved, for example, by their transportation inside the EVs which enables them to play a protective role under stress conditions at the cell wall or in matrix remodeling [28,55,111,119,156,157]. 

It was previously shown that some glycolytic enzymes can likely still retain the enzymatic activity related to their basic function in the cell even after forming complexes on a selected surface, e.g., the lipid membrane [158,159,160,161]. A role in energy production through glycolysis reactions conducted outside cells was previously suggested for bacteria, and this phenomenon may also make a significant contribution to bacterial virulence [11]. Two ATP molecules are produced during glycolysis and their local concentration outside cells may be beneficial for different microorganisms that are co-existing in a particular infectious niche. It was shown that the *C. parapsilosis* fungus demonstrates ecto-ATPase activity and thus the ability to hydrolyze extracellular ATP, and that this had a significant positive impact on the ability of these cells to adhere to epithelial cells [162]. The possibility cannot be excluded also that such glycolysis products or intermediates surrounding fungal cells are used as an energy source or for intercellular communication, which could also be beneficial during infection. However, it has been demonstrated also that extracellular ATP may play an important role in modulating the host immune response during fungal infection. For example, the extracellular ATP secreted by *C. albicans* augments the host defense via interactions with the purinergic receptors P2RX3 of epidermal MRGPRD+ (Mas-related G-protein coupled receptor D) nonpeptidergic neurons in the skin. This reduces the virulence of particular *C. albicans* strains that release higher amounts of extracellular ATP. However, the neuronal mediators involved in this phenomenon and that activate the host immune response against *C. albicans* have not yet been identified [163]. Another mechanism involved in the regulation of host responses to fungal infection via the extracellular ATP released by *C. albicans* is associated with the candidalysin-dependent triggering of the host innate immunity through processes related to the activation of epidermal growth factor receptor (EGFR) in the oral epithelial cells, followed by MAPK signaling and the production of inflammatory cytokines [164].

The evidence to date thus indicates that the presence of moonlighting proteins at the surface of *C. albicans* can have advantages and disadvantage for the host and it should be borne in mind there is an unceasing tilting of this balance one way or the other during infection. Not only can these proteins acquire the role of virulence factors, but, as it has been demonstrated repeatedly, some moonlighting proteins may stimulate host innate immunity, thus providing protection against fungal infections [165,166,167,168,169]. Several *C. albicans* surface-exposed proteins, including Eno1, Pgk1, Fba1, Pgi1, Tpi1, Gpm1, Cdc19, Adh1, Tdh3, Met6 and heat shock proteins of the Hsp70 family, have been identified as cell wall-associated moonlighting proteins that are immunoreactive during invasive fungal infection in humans. However, not all of the produced antibodies provided an evidently protective effect for the host and the presence of some might even correlate with an increased risk of a fatal outcome, such as anti-Tdh3 or anti-Ssb1 antibodies [170,171]. Furthermore, in other studies using a mouse model of disseminated candidiasis, it was demonstrated that antibodies against Cdc19 conferred protection against *C. albicans* in immunized mice, whereas anti-Fba1 did so against *C. glabrata* infection [71]. In *C. parapsilosis*, immunoreactive proteins were eukaryotic initiation factor 4A or translation initiation factor eIF4A subunit (Tif1), Atp2, Eno1, Tdh3, Pgk1, Adh1, Fba1, heat shock protein 70 Ssb1, pyruvate decarboxylase (Pdc11), Atp1, isocitrate dehydrogenase (Idh2) and guanine nucleotide-binding protein subunit beta-like protein (Bel1) [80]. In similar studies on *C. tropicalis*, Eno1, Fba1, Tdh3, Tif1, Atp1, Atp2, Bel1, Idh2, Cit1, ketol-acid reductoisomerase (Ilv5), Tpi1 and dihydrolipoyllysine-residue succinyltransferase (Kgd2) were indicated as cell wall-associated antigens [81]. 

The presence of IgG antibodies against moonlighting proteins in the blood of infected patients can be used to perform an accurate and fast diagnosis of invasive fungal infections, which is still a challenging issue. Such ELISA (enzyme-linked immunosorbent assay) tests have been proposed based on anti-Eno1, anti-Pgk1 and anti-Fba1 IgG identification and provide satisfactory sensitivity, specificity and positive and negative predictive values [41,172,173,174]. Given the often-reported possible protective role of antibodies to some of the aforementioned moonlighting proteins, numerous attempts have also been made to develop effective vaccines against infections caused by *C. albicans* using these proteins. In the mouse model of candidiasis, a vaccine containing Fba1 peptides was found to be capable of inducing humoral and cellular immune responses, thus providing protection against *C. albicans* infection [175,176]. A similar attempt to design a vaccine against *C. albicans* was also made for the Eno1 moonlighting protein [177,178]. However, such an approach failed in the case of *C. albicans* Tdh3 and Pgk1 and these vaccines proved to be ineffective [177,179]. In the case of infections caused by *C. tropicalis*, it was also demonstrated that immunization with a vaccine containing peptides derived from Met6 provided protection against disseminated candidiases in immunocompromised mice [180]. 

As moonlighting proteins at the fungal cell surface often perform their additional functions associated with fungal virulence on the basis of their co-existence at the same location, interactions and complementary activities, the design of anti-*Candida* vaccines should take this into account and make use of the abundant co-presence of these immunogenic proteins at the surfaces of pathogens to develop multivalent vaccines. These would likely provide more effective prevention against fungal infections in the future, particularly if combined with other cell wall component epitopes, including polysaccharides or covalently bound cell wall proteins. Care should always be taken, however, to avoid using epitopes, in vaccine design, that are shared between the pathogen and the host, an issue that may be particularly important when using evolutionarily conserved enzymes from basic metabolic cycles [181,182,183,184].

## 5. Concluding Remarks

**Figure 2:**
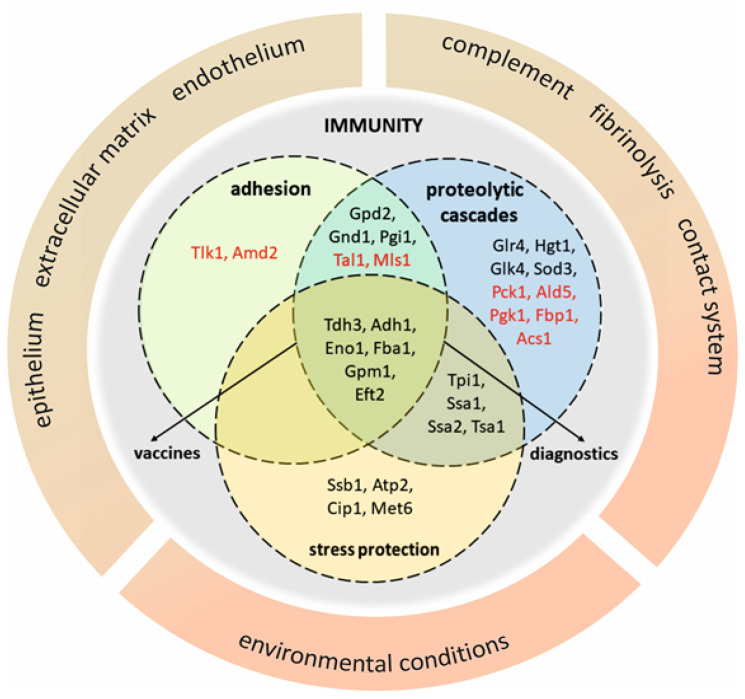
Diagram that groups moonlighting proteins on the basis of their involvement in adhesion to host cells and extracellular matrix proteins, activation of homeostatic/hemostatic proteolytic cascades and protection against stressful environmental conditions. The broad specificity actions are illustrated by the overlap of various functions performed by individual proteins, whilst the presence of at least several proteins in each circle emphasizes the collective action of moonlighting proteins that may be potential vaccine or diagnostic targets. Symbols of *C. albicans* moonlighting proteins are enriched with selected homologues of non-albicans *Candida* species (red font).

In *Candida* yeasts, some intracellular proteins emerge at the cell surface and become attached to the cell wall for a sufficient time to play key roles in pathogen adaptation to the environment within the human host organism. These “moonlighting” functions are sometimes difficult to precisely define and, for any individual fungal protein, their definitions usually rely on broad-specificity interactions with multiple molecular targets in the host (Figure 2). However, rather than an individual protein, distinct groups comprising at least several moonlighting proteins seem to contribute to particular phenomena that are essential for host colonization and infection, such as the direct adhesion to host cells and tissues, activation of host homeostatic proteolytic cascades, protection against environmental stress factors and molecular mimicry. Moreover, these moonlighting functions often overlap for significant numbers of fungal surface proteins.

The universality, low-specificity and multiplicity of the anticipated functions of these enigmatic proteins at the fungal surface prompt the hypothesis that moonlighting proteins act collectively to form a protective coat around the pathogenic cell, increasing the compatibility of its surface with host environment, neutralizing the actions of many stress factors and still contributing to immune evasion and the adherence to the host tissues. These joint actions are additionally supported by the formation of biofilm structures, in which known moonlighting proteins have been often detected. Moreover, some of these factors in freely soluble form may diffuse to the fungal cell microenvironment to exert effects at longer distances and promote the colonization of new niches by the pathogen and the spread of infection.

A hypothesis involving the collective and redundant action of candidal moonlighting proteins may explain why studies on the possible roles of individual surface proteins as virulence or pathogenicity factors often bring equivocal results, particularly when knockout mutants are used. On the other hand, the presence of numerous proteins on the microbe surface that were originally intracellular and thus specific for the pathogen offers opportunities for the accurate diagnosis of candidiasis, especially if sensitive antibody tests that combine the detection of several moonlighting proteins can be used. The co-existence of moonlighting proteins at the same location and the redundancy in their shared activities should also be taken into account while designing anti-*Candida* vaccines, which preferably should be multivalent to offer a chance for more effective prevention of fungal infections in the future.

## Figures and Tables

**Table 1:** Intracellular and extracellular functions of representative *C. albicans* moonlighting proteins.

Protein	Intracellular Function	Extracellular Function	Reference
Tdh3	catalysis/glycolysis pathway	ECM-binding,	[83]
		HPG-binding,	[77,84,85,86]
Eno1	catalysis/glycolysis pathway	adhesion to endothelial cells,	[28]
		HPG-binding,	[77,85,86]
		interaction with contact system proteins,	[73,87]
Gpm1	catalysis/glycolysis pathway	adhesion to endothelial cells and keratinocytes,	[29]
		ECM-binding,	[29]
		HPG-binding,	[84,88]
		interaction with component of complement system,	[88]
		interaction with contact system proteins,	[73,87]
		cleavage activity of C3b,	[88]
Adh1	catalysis/fermentation	ECM-binding,	[89]
		HPG-binding,	[77,84]
Gpd2	catalysis/carbohydrate metabolism	adhesion to endothelial cells and keratinocytes,	[90]
		ECM-binding,	[90]
		HPG-binding,	[77,90]
		interaction with component of complement system,	[90]
Ssa1	chaperoning	induction of endocytosis,	[45]
		Hst5-binding,	[42]
Eft2	protein synthesis	interaction with contact system proteins	[73,87]
